# Leucine-rich repeat containing 8A contributes to the expansion of brain ventricles in zebrafish embryos

**DOI:** 10.1242/bio.048264

**Published:** 2020-01-29

**Authors:** Yen-Tzu Tseng, Chun-Lin Ko, Chia-Teng Chang, Yen-Hua Lee, Wei-Chun Huang Fu, I-Hsuan Liu

**Affiliations:** 1 Department of Animal Science and Technology, National Taiwan University, Taipei, 106, Taiwan; 2 Graduate Institute of Cancer Biology and Drug Discovery, College of Medical Science and Technology, Taipei Medical University, Taipei, 110 Taiwan; 3 Research Center for Developmental Biology and Regenerative Medicine, National Taiwan University, Taipei, 106, Taiwan; 4 Department of Veterinary Medicine, School of Veterinary Medicine, National Taiwan University, Taipei, 106, Taiwan

**Keywords:** Volume-regulated anion channel (VRAC), Volume-sensitive organic osmolyte/anion channel (VSOAC), Lrrc8A, Taurine, Organic osmoregulation

## Abstract

The sodium osmotic gradient is necessary for the initiation of brain ventricle inflation, but a previous study predicted that organic and inorganic osmolytes play equivalently important roles in osmotic homeostasis in astrocytes. To test whether organic osmoregulation also plays a role in brain ventricle inflation, the core component for volume-regulated anion and organic osmolyte channel, *lrrc8a*, was investigated in the zebrafish model. RT-PCR and whole-mount *in situ* hybridization indicated that both genes were ubiquitously expressed through to 12 hpf, and around the ventricular layer of neural tubes and the cardiogenic region at 24 hpf. Knocking down either one *lrrc8a* paralog with morpholino oligos resulted in abnormalities in circulation at 32 hpf. Morpholino oligos or CRISPR interference against either paralog led to smaller brain ventricles at 24 hpf. Either *lrrc8aa* or *lrrc8ab* mRNA rescued the phenotypic penetrance in both *lrrc8aa* and *lrrc8ab* morphants. Supplementation of taurine in the E3 medium and overexpression *csad* mRNA also rescued *lrrc8aa* and *lrrc8ab* morphants. Our results indicate that the two zebrafish *lrrc8a* paralogs are maternal message genes and are ubiquitously expressed in early embryos. The two genes play redundant roles in the expansion of brain ventricles and the circulatory system and taurine contributes to brain ventricle expansion via the volume-regulated anion and organic osmolyte channels.

## Abstract (precis)

**Summary:** This study provides evidence to demonstrate that organic osmolytes such as taurine contribute to the inflation of the brain ventricle via the volume-sensitive organic osmolyte-anion channel during zebrafish embryogenesis.

## INTRODUCTION

The regulation and homeostasis of osmolality is a critical mechanism for all active cells as biological activities are generally in an aqueous environment with various organic and inorganic solutes. In general, cells influx osmolytes to trigger a regulatory volume increase (RVI) in hyper-osmotic environments, whereas efflux osmolytes trigger regulatory volume decreases (RVD) in a hypo-osmotic environment. The existence of a volume-regulated anion channel (VRAC) was first putatively proposed based on the observations of an outwardly rectifying Cl^−^ current and consequently an RVD when cells were exposed to hypotonic solutions (Cahalan and Lewis, 1988; Hazama and Okada, 1988). Later, organic osmolytes such as taurine were considered accountable for at least half of the total RVD, and hence a putative volume-sensitive organic osmolyte/anion channel (VSOAC) was proposed for this activity (Garcia-Romeu et al., 1991; Jackson and Strange, 1993; Strange and Jackson, 1995). Due to the pharmacological similarity and controversial experimental results, the question of whether VRAC is identical to VSOAC was once a highly debated issue (Díaz et al., 1993; Kirk and Kirk, 1994; Lambert and Hoffmann, 1994; Sanchez-Olea et al., 1995; Shennan et al., 1994).

Despite the extensive studies on the properties of this channel for over 20 years, the molecular identity of this channel remained completely elusive until 2014. Two independent groups used a similar strategy and identified leucine-rich repeat containing 8A (LRRC8A) as an indispensable component in constituting VRAC in HEK293 cells (Qiu et al., 2014; Voss et al., 2014). In the human genome, the LRRC8 gene family contains five genes, including LRRC8A to LRRC8E, and shares some homology with pannexins, which constitute hexameric channels (Abascal and Zardoya, 2012). Current evidence suggests that the assembly of LRRC8A and LRRC8D recapitulates all the features of VSOAC, while the LRRC8A and other members of LRRC8 family (LRRC8B, LRRC8C and LRRC8E) mimic most of the features of VRAC except for the taurine efflux (Planells-Cases et al., 2015).

Osmoregulation has been shown to play critical roles in morphogenesis during embryonic development. For example, the cavitation of blastocysts requires the activity of a sodium pump (Wiley, 1984; Burgener-Kairuz et al., 1994). Additionally, the inflation of cerebral ventricles also requires the activity of a sodium pump (Lowery and Sive, 2005). Organic and inorganic osmolytes play equivalently important roles in osmotic homeostasis in astrocytes (Pasantes-Morales et al., 1993). Despite the demonstration that both polyols (such as inositol or sorbitol) and several amino acids and their derivatives (such as aspartate and glutamate) participate in RVD, possibly via VRAC/VSOAC (Banderali and Roy, 1992; Jackson and Strange, 1993), taurine has been the most epitomic organic osmolyte released via VRAC/VSOAC. Previous studies indicate that both *taut*, the gene coding for the transporter for cellular taurine intake, and *csad*, the gene coding for the key enzyme for taurine *de novo* synthesis, are maternal messages in zebrafish embryos (Chang et al., 2013; Kozlowski et al., 2008). A recent study shows two *lrrc8a* genes in the zebrafish genome, and both protein products act identically to the human LRRC8A protein in VRAC (Yamada et al., 2016). Interestingly, knockdown of either *lrrc8aa* or *csad* produces a similar cardiac abnormality (Yamada et al., 2016; Chang et al., 2013). In this study, we aimed to characterize the roles of two paralogous *lrrc8a* genes in embryonic development and test the hypothesis that Lrrc8a also contributes to the formation and inflation of brain ventricles by modulating organic osmolytes during embryogenesis.

## RESULTS

### Zebrafish has two *lrrc8a* genes

**Fig. 1.**
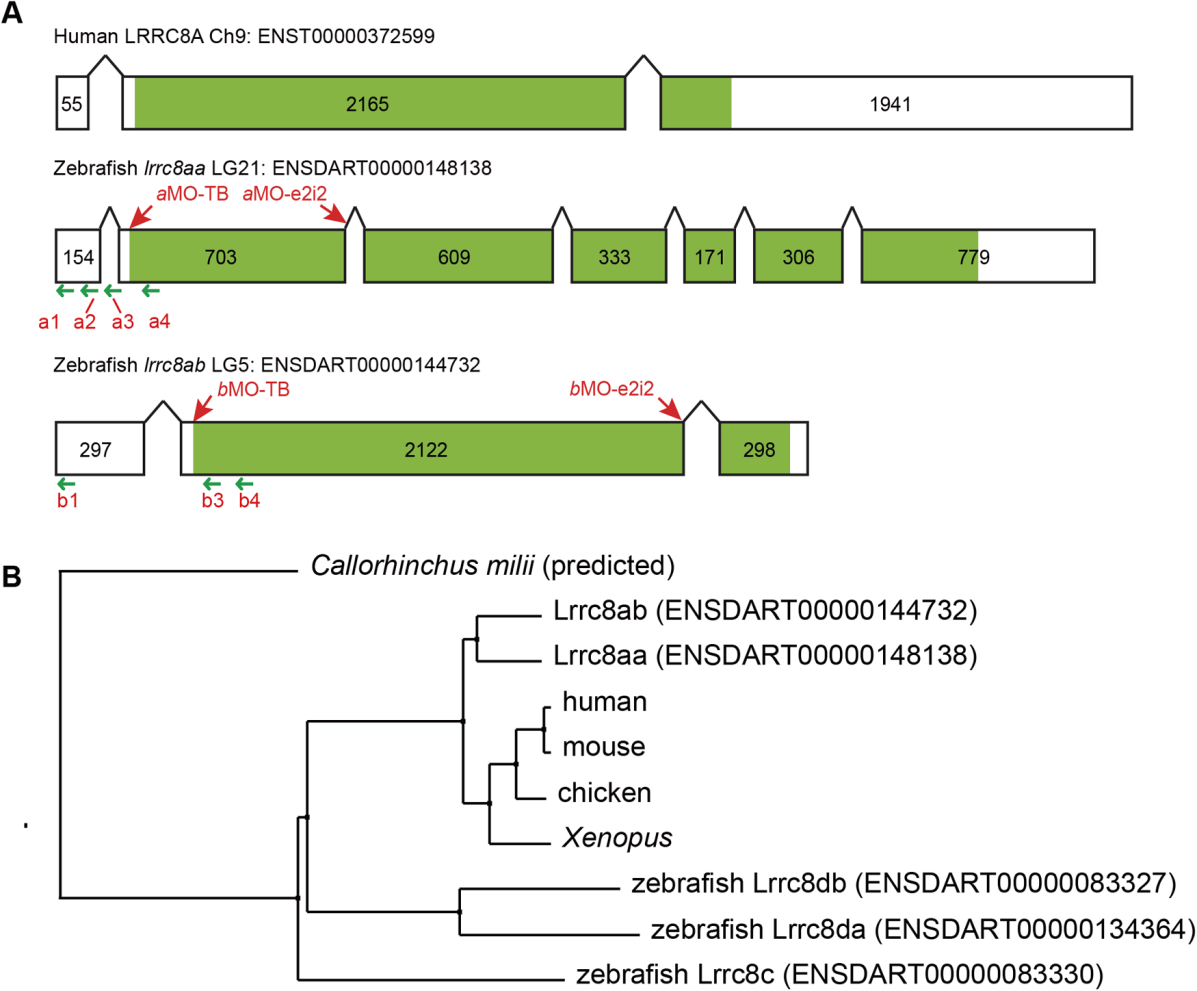
**Zebrafish have two *lrrc8a* paralogs.** (A) Two homologous human LRRC8A genes were found in the zebrafish genome (*lrrc8aa* or *swell1a*, ENSDART00000148138; and *lrrc8ab* or *swell1b*, ENSDART0000144732), but only *lrrc8ab* has a similar genomic structure to mammalian *LRRC8A.* The exons are presented as boxes and the coding regions are shaded in green. MOs target sites are indicated by red arrows. CRISPRi guide RNA target sites are indicated by green arrows. (B) Phylogenetic tree constructed by neighbor-joining method with percentage identity distances indicating that both Lrrc8a paralogs are highly conservative throughout evolution. Lrrc8c and two Lrrc8d paralogs are also well-aligned in the phylogenetic tree. The predicted Lrrc8a protein sequence in *Callorhinchus milii* (Australian ghostshark, a cartilaginous fish) was used as an outgroup.

We confirmed that there were two genes in the zebrafish genome with protein coding sequences similar to human LRRC8A and these have been annotated as *lrrc8aa* (ENSDART00000148138) and *lrrc8ab* (ENSDART0000144732). Despite the similar amino acid sequences of zebrafish Lrrc8aa and Lrrc8ab compared to mammalian LRRC8A, only *lrrc8ab* has a similar genomic structure to human *LRRC8A* (Fig. 1A)*.* Phylogenetic tree construction (Waterhouse et al., 2009) using a neighbour-joining method with percentage identity distances showed that both Lrrc8aa and Lrrc8ab are highly conserved throughout evolution (Fig. 1B). Interestingly, one of the LRRC8 family members, *lrrc8b*, was not found in the zebrafish genome, whereas two *lrrc8d* paralogs, annotated as *lrrc8da* and *lrrc8db*, were identified.

**Fig. 2.**
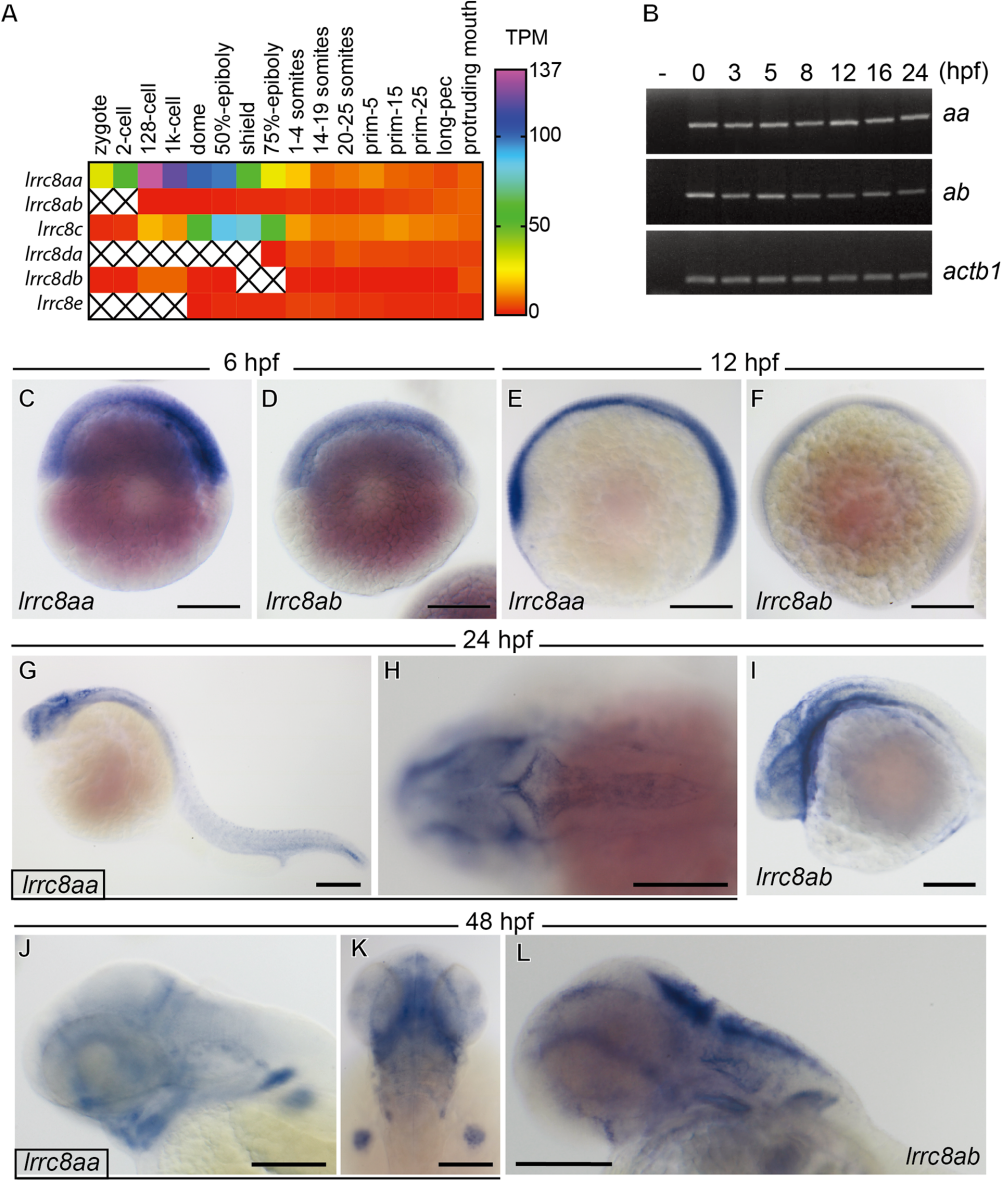
**The expression profiles of zebrafish *lrrc8aa* and *lrrc8ab.*** (A) The expression levels of *lrrc8* family during zebrafish embryogenesis in transcripts per million (TPM) were obtained from an Expression Atlas. *Lrrc8aa* is much more abundant than *lrrc8ab* in the first 24 h of development. While *lrrc8c* is the most abundant family member during gastrulation; the expression levels are comparable after 24 hpf (prim-5). (B) The sequencing result did not detect *lrrc8ab* in zygotes and at the two-cell stage, but RT-PCR with β-actin (*actb1*) as the loading control showed that both *lrrc8a* genes are maternal messages and can be detected at all developmental stages through to 72 hpf. (C–L) Whole-mount *in situ* hybridization of *lrrc8aa* (C,E,G,H,J,K) and *lrrc8ab* (D,F,I,L) showed that both genes are ubiquitously presented in the zebrafish embryo at 6 (C,D) and 12 (E,F) hpf. In 24 hpf embryos (G–I), both genes could be clearly detected at the ventricular layer of the brain as well as the cardiogenic region. In 48 hpf embryos (J–L), both genes could be detected at brain ventricles, retina, otic vesicles and pectoral fin buds. Scale bars: 200 µm.

The quantitative expression profiles of the lrrc8 family during zebrafish embryogenesis can be retrieved in the published dataset (Fig. 2A) (White et al., 2017). As previously described (Yamada et al., 2016), *lrrc8aa* is much more abundantly expressed during the first 24 h of zebrafish embryogenesis (Fig. 2A). Among other family members, *lrrc8c* is the most abundantly expressed gene and the expression of both *lrrc8da* and *lrrc8db* were detectable after gastrulation (Fig. 2A). To validate this result, RT-PCR was performed with β-actin (*actb1*) as the loading control (Fig. 2B). Interestingly, both *lrrc8aa* and *lrrc8ab* were detected in the freshly laid embryos (Fig. 2B). The PCR products of both genes can be detected at all developmental stages through 72 hours post fertilization (hpf) indicating temporally ubiquitous expressions (Fig. 2B). Whole-mount *in situ* hybridization further demonstrated spatially ubiquitous expression of both *lrrc8aa* and *lrrc8ab* in the early embryos (Fig. 2C–F). In 24 hpf embryos, both *lrrc8aa* (Fig. 2G,H) and *lrrc8ab* (Fig. 2I) are prominently expressed at the ventricular layer along the brain, as well as the cardiogenic region. In 48 hpf embryos, both genes can be detected in brain ventricles, retina, otic vesicles and pectoral fin buds (Fig. 2J,K,L). Interestingly, the expressions of both genes seem predominantly overlapped, suggesting that the two *lrrc8a* genes in zebrafish might play redundant roles during early embryogenesis.

### Both *lrrc8aa* and *lrrc8ab* contributed to the development of circulatory system

**Fig. 3.**
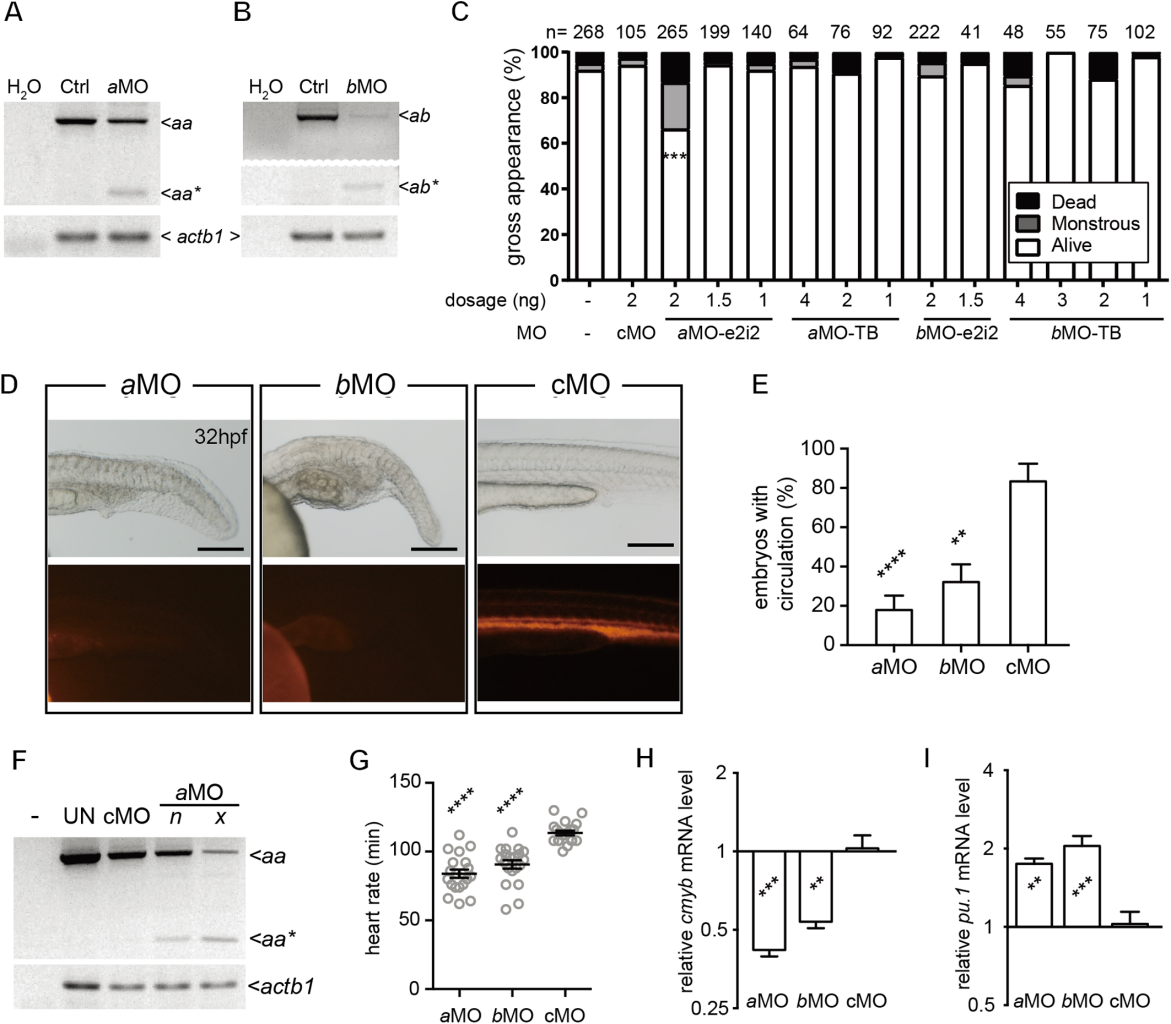
**Reduction of either one of the *lrrc8a* paralogs resulted in identical abnormalities in the circulatory system.** (A,B) RT-PCR showed that the splicing of both *lrrc8aa* (A) and *lrrc8ab* (B) can be perturbed by the respective e2i2-MOs and result in a shorter transcript (aa* in A; ab* in B). Sequencing of the PCR products indicated that exon 2 of both genes was missing. (C) Injection of 2 ng *lrrc8aa-*MO e2i2 led to significantly increased embryonic death and severe malformations. The sample size (*n*) of each group is indicated on top of the histogram. (D) Knockdown of either *lrrc8aa* (*lrrc8aa*-MO e2i2) or *lrrc8ab* (*lrrc8ab*-MO e2i2) resulted in accumulation of posterior blood islands and impaired the expansion of circulation to this region at 32 hpf. Scale bars: 200 µm. (E) The ratios of embryos with blood circulation were reduced in *lrrc8aa*-MO e2i2 and *lrrc8ab*-MO e2i2 morphants. (F) Correlation between gene knockdown efficacy (*lrrc8aa-*MO e2i2) and phenotypically normal (*n*) or not (*x*) was demonstrated with RT-PCR. -, no sample control; UN, untreated control. (G) Heart rates in both *lrrc8aa-*MO e2i2 and *lrrc8ab-*MO e2i2 morphants were significantly lower compared to the control morphants at 32 hpf. (H,I) At 32 hpf, quantitative RT-PCR showed that both the hematopoietic stem cell marker, *cmyb*, and the myeloid cell marker, *pu.1*, were significantly altered in both *a*MO and *b*MO morphants when compared to control morphants. *a*MO, *lrrc8aa*-MO e2i2; *b*MO, *lrrc8ab*-MO e2i2; cMO, control MO. **P<0.01; ***P<0.001; ****P<0.0001.

To investigate the physiological roles of *lrrc8aa* and *lrrc8ab* during zebrafish embryogenesis, two exon-intron boundary-targeted antisense morpholino oligos (MOs), *lrrc8aa*-MO e2i2 (*a*MO-e2i2) and *lrrc8ab*-MO e2i2 (*b*MO-e2i2) as well as two translation-blocking MOs, *lrrc8aa*-MO TB (*a*MO-TB) and *lrrc8ab*-MO TB (*b*MO-TB) (Fig. 1A) were designed. To confirm the perturbation of mRNA splicing, RT-PCR flanking exon 1 to 3 of both *lrrc8a* genes showed that *a*MO-e2i2 and *b*MO-e2i2 effectively disrupted the splicing of *lrrc8aa* (aa*, Fig. 3A) and *lrrc8ab* (ab*, Fig. 3B) mRNA, respectively. Cloning and sequencing of the mis-spliced RT-PCR products showed that both MOs led to the elimination of entire exon 2s, which contain the start codons for both genes and hence their protein products were presumably knocked-down.

The ratio of normal embryos was significantly lower only in 2 ng of *a*MO-e2i2 morphants (176/265, *P*<0.001) compared to embryos injected with 2 ng of control MO (cMO, 182/193). When compared to control morphants, the other MOs and tested doses did not significantly cause more death or monstrous embryos, in which developmental abnormalities were too severe to classify. (Fig. 3C). In addition, we also observed the pericardial effusion phenotype (data not shown) as previously described (Yamada et al., 2016). Furthermore, accumulation of the posterior blood island (PBI) at 32 hpf was constantly observed (Fig. 3D). Angiography of 32 hpf morphants indicated that both 2 ng of *a*MO-e2i2 (5/28, *P*<0.0001) or *b*MO-e2i2 (9/28, *P*<0.01) significantly perturbed the normal blood circulation compared to control morphants (15/18) (Fig. 3E). Among the alive non-monstrous morphants, RT-PCR clearly demonstrated a higher level of normal *lrrc8aa* transcripts (aa) and less splicing-perturbed transcript (aa*) in *a*MO-e2i2 morphants that appeared to be completely normal (*n* in Fig. 3F) when compared to morphants with circulatory phenotypes (*x* in Fig. 3F), such as pericardial effusion and PBI accumulation. Furthermore, among the alive non-monstrous morphants, the survival rate (relative to 24 hpf) was significantly lower in the phenotypic (cardiac effusion and PBI accumulation) morphants (48 hpf: 25/38 in 2 ng of *a*MO-e2i2 morphants, *P*<0.05; 27/40 in 2 ng of *b*MO-e2i2 morphants, *P*<0.0001) compared to the morphants with normal appearance (41/45 in 2 ng of *a*MO-e2i2 morphants; 61/67 in 2 ng of *b*MO-e2i2 morphants).

The heart rate (113.5/min in cMO, *n*=20) was also significantly decreased in 2 ng of *a*MO-e2i2 (83.9/min, *n*=20, *P*<0.0001) and 2 ng of *b*MO-e2i2 (90.7/min, *n*=20, *P*<0.0001) morphants at 32 hpf (Fig. 3G). Previous studies indicate that PBI contributes to the third wave of hematopoiesis after 26 hpf (Medvinsky et al., 2011) and hemodynamics is a critical factor for the differentiation of hematopoietic stem cells (North et al., 2009). Accordingly, quantitative RT-PCR showed that the hematopoietic stem cell marker *cmyb* was significantly decreased, whereas the myeloid lineage marker *pu.1* was significantly increased in *a*MO (*cmyb:* 0.419, *P*<0.001; *pu.1:* 1.749, *P*<0.01; *n*=5) and *b*MO (*cmyb:* 0. 537, *P*<0.01; *pu.1:* 2.047, *P*<0.001; *n*=5) morphants when compared to the control morphants (*cmyb:* 1.028; *pu.1:* 1.028; *n*=5) (Fig. 3H,I).

### Both *lrrc8aa* and *lrrc8ab* contributed to the morphogenesis of brain ventricle

**Fig. 4.**
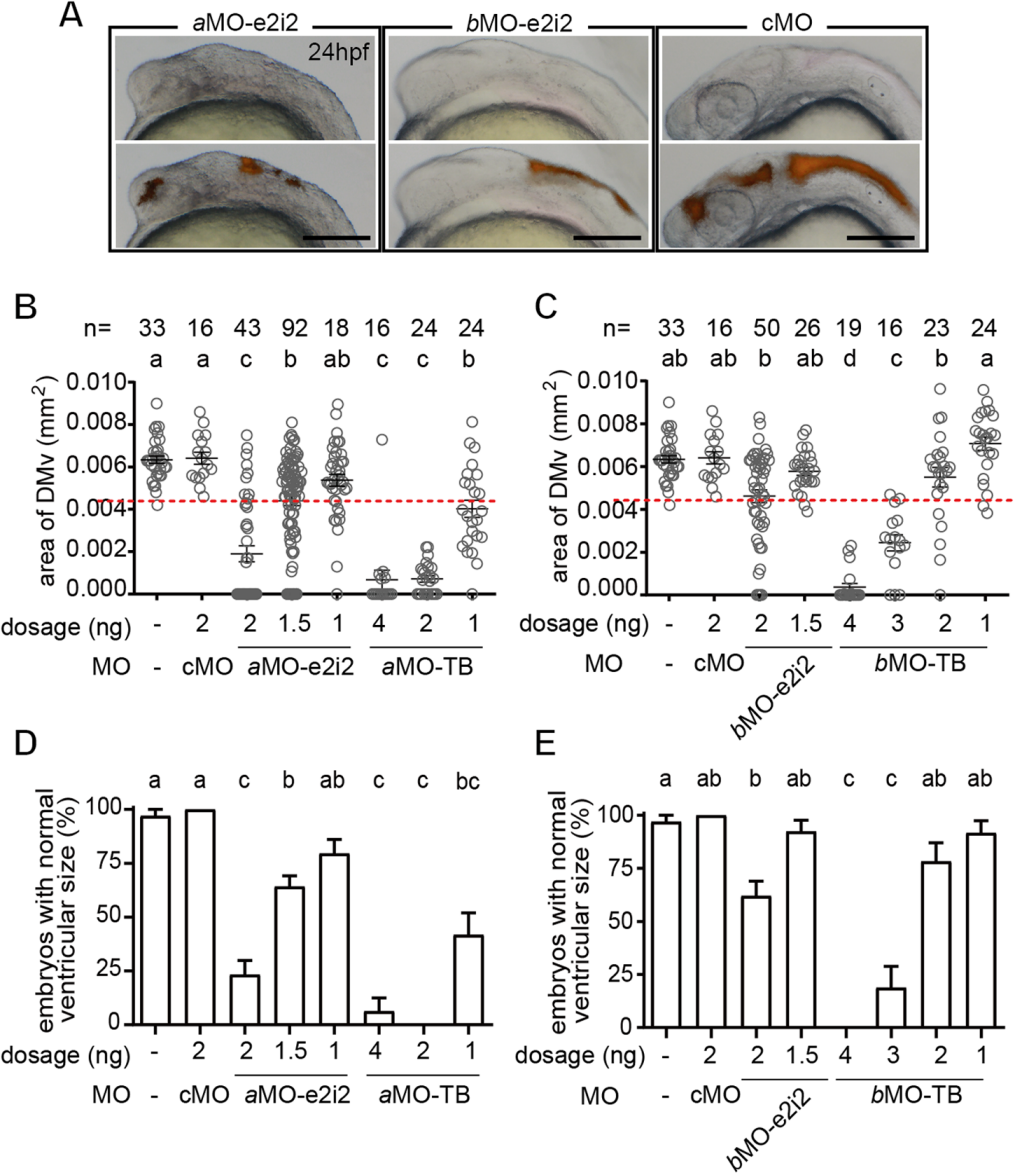
**Knockdown of either zebrafish *lrrc8a* paralog perturbed the inflation of the brain ventricle.** (A) The inflation of the brain ventricle was impaired in *lrrc8a* morphants. Scale bars: 200 µm. (B,C) The DMv area size was significantly reduced in *lrrc8aa-*MO e2i2 (*a*MO) and *lrrc8ab-*MO e2i2 (*b*MO) morphants compared to untreated embryos and control morphants (cMO). The sample size (*n*) of each group is indicated on top of the histogram. (D,E) The mean value minus two standard deviations of the untreated embryos was defined as the lower normal boundary (4.3507, red dotted line in B,C). Both *lrrc8aa-*MO e2i2 and *lrrc8ab-*MO e2i2 resulted in significantly lower normal rate compared to the untreated embryos and control morphants. Different letters on top of the histograms represent different statistical groups (*P*<0.05).

To elucidate the possible role of *lrrc8a* genes in the formation of brain ventricles, the same fluorescent TRITC-dextran (20 mg/ml) used for angiography was injected into the newly formed fourth brain ventricle in alive non-monstrous 24 hpf zebrafish embryos. The overlaid micrographic images clearly depicted smaller ventricular areas in the *a*MO-e2i2 and *b*MO-e2i2 morphants compared to the control morphants (Fig. 4A).

To further characterize the abnormality in brain ventricle, 24 hpf morphants or untreated embryos were documented and the area representing the diencephalic/mesencephalic ventricle (DMv) was measured in ImageJ (Fig. 4B,C). Injection of every *lrrc8a* MO resulted in smaller DMv area compared to control morphants and untreated controls dose-dependently (Fig. 4B,C). The Shapiro-Wilk normality test indicated that the data for DMv of untreated controls are a normal distribution (*P*=0.5706). Statistically, the range of two standard deviations from the mean should include more than 95% of the population with a normal distribution. To calculate the phenotypic penetrance, we therefore arbitrarily defined any DMv area with a size smaller than the mean of the untreated control (0.006339 mm^2^) minus two standard deviations (2×0.0009943) as significantly smaller (∼0.004350 mm^2^) and phenotypically abnormal. The result showed that, among the alive and non-monstrous embryos, the injection of *lrrc8a* MOs dose-dependently caused increased abnormal rate compared to the cMO (Fig. 4D,E).

**Fig. 5.**
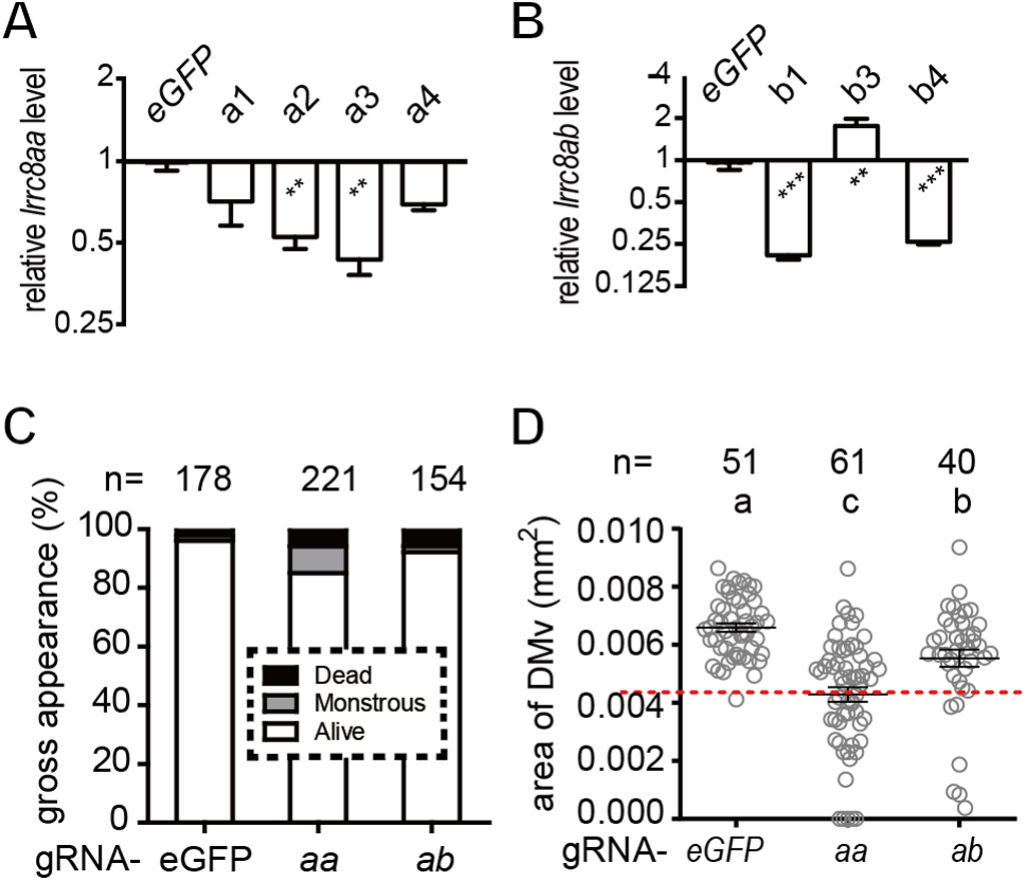
**Knockdown of both zebrafish *lrrc8a* paralogs by CRISPRi.** (A,B) Quantitative RT-PCR showed that injection of dCas9 mRNA with certain *lrrc8aa* (a2 and a3) or *lrrc8ab* (b1 and b4) gRNA significantly reduced the respective transcript compared to the group with dCas9 and eGFP gRNA. (C,D) A mixture of gRNA against *lrrc8aa* (*aa*) or *lrrc8ab* (*ab*) did not cause increased embryonic death and severe malformations (C), but showed significantly smaller DMv area size (D). Each alphabetical letter above the histograms denotes a distinct statistical group (*P*<0.05). The lower normal boundary is shown as red dotted line. The sample size (*n*) of each group is indicated on top of the histogram. **P<0.01, ***P<0.001.

To further validate the specificity of this smaller ventricle phenotype in two sets of *a*MO and *b*MO morphants, CRISPRi was used to knockdown the transcriptions of *lrrc8aa* and *lrrc8ab* mRNA. A pool of gRNA targeting the non-template strand near the transcriptional/translational starting sites was selected from CRISPRscan (Moreno-Mateos et al., 2015) (Fig. 1A). The mRNA encoding the catalytically de-activated Cas9 (dCad9) was co-injected with each gRNA targeting *lrrc8aa* or *lrrc8ab* with gRNA targeting green fluorescent protein (gRNA-*eGFP*) as control (Liao et al., 2015; Shalem et al., 2014). Quantitative real-time PCR indicated that two of the gRNA against *lrrc8aa* (*a2* and *a3* in Fig. 5A) and *lrrc8ab* (*b1* and *b4* in Fig. 5B) effectively reduced the targeted genes. With the absence of significant gross phenotype (Fig. 5C), the mixture of these gRNA targeting *lrrc8aa* (gRNA-*aa*, *P*<0.0001) or *lrrc8ab* (gRNA-*ab*, *P*=0.009) indeed significantly reduced the area size of DMv compared to the gRNA-eGFP control (Fig. 5D). However, the phenotypic penetrance was significantly observed only in the gRNA-*aa* group (28/61, *P*<0.0001), but not in gRNA-*ab* group (6/40) compared to the gRNA-*eGFP* control (1/51) suggesting that *lrrc8ab* might play a relatively minor role in ventricular morphogenesis compared to *lrrc8aa*.

### *Lrrc8aa* and *lrrc8ab* play a redundant role in brain ventricle inflation

**Fig. 6.**
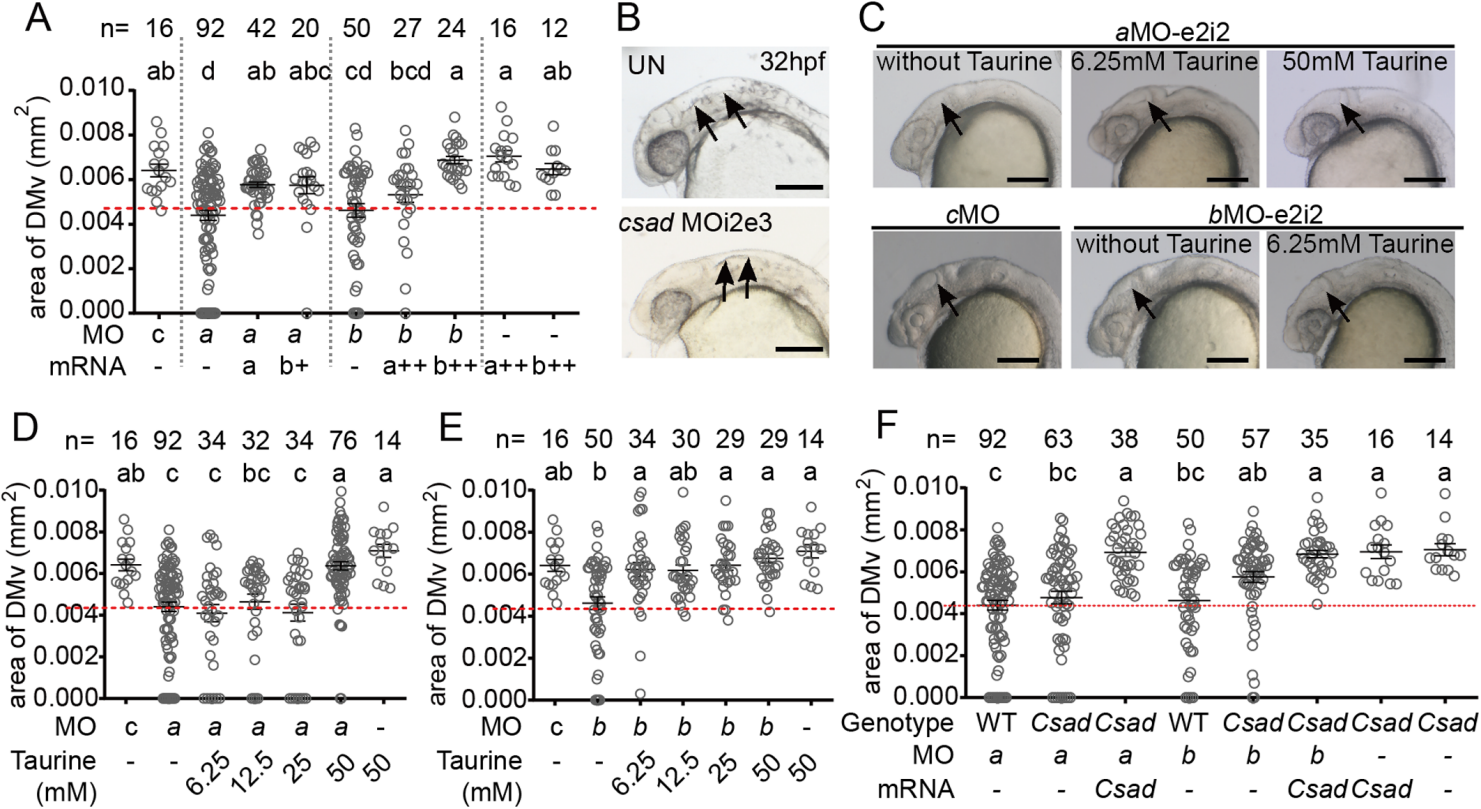
***Lrrc8aa* and *lrrc8ab* play redundant roles in facilitating the inflation of the brain ventricle.** (A) The smaller DMv phenotype in *lrrc8aa*-MO e2i2 or *lrrc8ab*-MO e2i2 morphants can be rescued by overexpression of either of the *lrrc8a* paralog mRNA. (B) The *csad*-MO i2e3 morphants show a smaller brain ventricle phenotype compared to the untreated embryo at 32 hpf. The arrows indicate the brain ventricle. Scale bars: 200 µm. (C–E) The smaller DMv phenotype in *lrrc8aa*-MO e2i2 or *lrrc8ab*-MO e2i2 morphants can be rescued by culture in embryo medium supplement with taurine. The DMv area size was increased in morphant culture with taurine (C). The images are representative median values of the DMv area of each group. Arrows indicate DMv area. Scale bars: 200 µm. The *lrrc8aa*-MO e2i2 (D) or *lrrc8ab*-MO e2i2 (E) morphants were cultured in embryo medium supplement with different amounts of taurine. The smaller DMv phenotype of *lrrc8aa*-MO e2i2 or *lrrc8ab*-MO e2i2 morphants could be rescued by supplementation of 50 mM or 6.25 mM taurine in embryo medium, respectively. (F) The *lrrc8a* MOs were injected into the *csad* transgenic embryos. The smaller DMv phenotypes were partially rescued in the *csad* transgenic background compared to the wild-type background. Overexpression of *csad* mRNA into *csad* transgenic embryo could fully rescued the smaller DMv phenotype. Different letters on top of the histograms represent different statistical groups (*P*<0.05). The lower normal boundary is shown as a red dotted line. The sample size (*n*) of each group is indicated on top of the histogram. cMO, control MO; *a*MO, *lrrc8aa*-MO e2i2; *b*MO, *lrrc8ab*-MO e2i2; mRNA-a, *lrrc8aa*-*IRES-eGFP* mRNA 100 pg; mRNA-a++, *lrrc8aa*-*IRES-eGFP* mRNA 200 pg; mRNA-b+, *lrrc8ab*-*IRES-eGFP* mRNA 150 pg; mRNA-b++, *lrrc8ab*-*IRES-eGFP* mRNA 200 pg; untreated embryo, UN; WT, wild-type; mRNA-*Csad*, *Csad*-*IRES-eGFP* mRNA 150 pg.

To further confirm that the phenotypes observed in *lrrc8a* morphants were due to the decrease of the corresponding *lrrc8a* gene products, *eGFP*, *lrrc8aa-IRES-eGFP* or *lrrc8ab-IRES-eGFP* mRNA was injected with or without *lrrc8a* MOs. The mRNA used in this experiment did not result in any abnormality in brain ventricle when injected alone. In *a*MO-e2i2 morphants, the areas of DMv were significantly rescued with the addition of *lrrc8aa-IRES-eGFP* (a: 100 pg in Fig. 6A) as well as *lrrc8ab-IRES-eGFP* (b+: 150 pg in Fig. 6A). On the other hand, the addition of *lrrc8aa-IRES-eGFP* and *lrrc8ab-IRES-eGFP* mRNA partially and fully rescued the small DMv phenotype resulting from *b*MO-e2i2, respectively (*b*MO with a++ and b++ mRNA in Fig. 6A; a++: 200 pg, b++: 200 pg). The *lrrc8aa* or *lrrc8ab* mRNA reciprocally rescued the small DMv phenotype caused by the other paralogous *lrrc8a* gene (Fig. 6A) suggesting that these two genes act in a redundant fashion in the morphogenesis of brain ventricle.

LRRC8A is an indispensable component of VRAC (Qiu et al., 2014; Voss et al., 2014), which mediates organic osmolytes such as taurine to efflux, while taurine efflux has long been a signature for VRAC/VSOAC activity (Jackson and Strange, 1993; Lambert and Hoffmann, 1994; Shennan et al., 1994). Our previous study showed that the homeostasis of taurine in zebrafish embryos predominantly depends on *de novo* synthesis via *csad* (Chang et al., 2013). Reduced *csad* level resulted in pericardial effusion, which is similar in *lrrc8a* morphants reported previously and in this study (Yamada et al., 2016), and can be rescued by taurine supplementation (Chang et al., 2013). Additionally, embryonic taurine deficiency also led to PBI accumulation (unpublished data) and smaller brain ventricle (Fig. 6B), it is tempting to speculate that both *lrrc8a* genes contribute to ventricular inflation via VRAC activity by modulating the distribution of organic osmolytes such as taurine.

To test whether an organic osmolyte such as taurine plays a role in *lrrc8a*-mediated brain ventricle inflation, taurine was supplemented to the embryo medium to rescue the phenotypes of *lrrc8a* morphants. Indeed, supplementation of 6.25 mM taurine in the embryo medium successfully rescued the small brain ventricle phenotype in *b*MO morphants (Fig. 6C,E). Interestingly, although the same concentration of taurine failed to rescued *a*MO morphants, increasing the supplementation concentration to 50 mM successfully rescued the small brain ventricle phenotype in *a*MO morphants (Fig. 6C,D).

To further validate the role of taurine in brain ventricle inflation, a transgenic zebrafish line that ubiquitously overexpress *csad* was generated and *lrrc8a* or *lrrc8b* MOs were injected into these *csad* transgenic embryos. Consistent with the taurine supplementation, morphants with *csad* transgenic background moderately resist the small brain ventricle phenotype induced by *lrrc8a* or *lrrc8b* MOs compared to the wild-type background (Fig. 6F). Co-injection of *Csad-IRES-eGFP* mRNA with *lrrc8a* or *lrrc8b* MOs fully rescued the small DMv phenotype (Fig. 6F) indicating that taurine participates in the brain ventricle inflation via VRAC/VSOAC during zebrafish embryogenesis.

## DISCUSSION

VRAC/VSOAC is currently considered constituted by LRRC8 family members with Lrrc8a as the indispensable core component (Qiu et al., 2014; Voss et al., 2014). We confirmed that there are two paralogous *lrrc8a* genes in the zebrafish genome (Yamada et al., 2016). The human chromosome 9 (Hsa9) includes multiple putative orthologs to the zebrafish linkage group 5 (LG5) and LG21 (Postlethwait et al., 2000). The loci of the zebrafish *lrrc8aa* on LG21 and *lrrc8ab* on LG5 were not evident in the previously reported locus of the human LRRC8A on Hsa9 (q34.11). The genomic structures of all human LRRC8 family members are similar to zebrafish *lrrc8ab* that the majority of the coding sequence is included within a single large exon; this was not the case for *lrrc8aa* (Fig. 1A). As a previous investigation suggests orthologs tend to retain similar genome structures (Xu et al., 2012), zebrafish *lrrc8ab*, compared to *lrrc8aa*, is probably evolutionarily closer to human LRRC8A.

We failed to find *lrrc8b* paralog in the zebrafish genome but two *lrrc8d* paralogs were found. Additionally, both *lrrc8db* and *lrrc8c* are located on LG6 immediately next to each other, whereas human LRRC8B, LRRC8C and LRRC8D locate on Hsa1 close to one another. Firstly, contrary to the proposal that LRRC8A and E arose by duplicating LRRC8B and C (Abascal and Zardoya, 2012), it is possible that LRRC8B was derived from *lrrc8c* or *lrrc8d* paralogs later in evolution. Secondly, as only LRRC8D is considered to contribute to VSOAC activity together with the essential channel component LRRC8A (Planells-Cases et al., 2015), the fact that only these two LRRC8 family members retain duplications in zebrafish might suggest the importance of organic osmoregulation in aquatic vertebrates such as zebrafish.

It is believed that, although polyploidy due to gene/genome duplication might be important for speciation and diversification, evolution tends to lead its way back to the diploid state through gene silencing and loss unless the duplicated genes are somehow mutated to introduce differences in temporal/spatial expressions or biochemical functions between paralogs (Gu et al., 2002; McLysaght et al., 2002; Force et al., 1999). The evidence provided previously and in this report demonstrate that *lrrc8aa* and *lrrc8ab* not only have similar temporal and spatial expression patterns (Fig. 2) and identical biochemical and cellular activities (Yamada et al., 2016), but also have similar biological function, as knocking down either one of the genes phenocopies the knockdown of the other (Figs 3–5). The overexpression of either gene can rescue the other one (Fig. 6A). In addition to expression and function, a later theory suggests that gene expression dosage might also be a reason for the retention of duplicated genes (Adams et al., 2003). Therefore, it is likely that the sum of total expression dosages of both *lrrc8a* paralogs is critical for early embryogenesis in zebrafish. Accordingly, as *lrrc8aa* is more abundantly expressed than *lrrc8ab*, the results of our knockdown and rescue experiments suggest that *lrrc8aa* plays a more dominant role than *lrrc8ab* (Fig. 6A)*.* Moreover*,* the smaller brain ventricle phenotypes in *lrrc8aa* morphants could only be rescued by a higher dose of taurine supplementation than in *lrrc8ab* morphants (Fig. 6A). However, we cannot exclude the possibility that *lrrc8aa* and *lrrc8ab* began to express differently in specific tissues and cells later in the development or in the adult zebrafish.

Consistent with the previous report (Yamada et al., 2016), the most observable phenotype in *lrrc8aa* or *lrrc8ab* knocked-down morphants was pericardial effusion, that can be seen after about 28 hpf (data not shown). In addition, many of these morphants were defective in the extension of blood circulation (Fig. 3D). In principle, hemodynamics were modulated by two major factors: the distribution of blood volume and the regulation on the circulation such as by cardiac output. Although the initiation of both cardiogenesis and vasculogenesis seems to be genetically programmed (Isogai et al., 2003; Lawson and Weinstein, 2002; Buckingham et al., 2005; Liao et al., 2008), it is clear that hemodynamic force feeds back and affects the continuation of cardiogenesis and vasculogenesis (Broekhuizen et al., 1999; Kowalski et al., 2012). In our study, we observed a significantly slower heart rate in both *lrrc8aa* morphants and *lrrc8ab* morphants. Since we did not observe significant alteration in early cardiac development markers such as *cmlc2* at 24 hpf *lrrc8aa* morphants (data not shown), it is likely that *lrrc8aa* or *lrrc8ab* are not required for initial cardiogenesis.

The previous studies suggest that the formation of plasma during embryonic vasculogenesis and angiogenesis is via the formation and fusion of intracellular vacuoles (Kamei et al., 2006; Folkman and Haudenschild, 1980). As the fundamental driving force for the vacuole formation is not well understood, it is intriguing whether organic osmoregulation and VRAC contribute to the formation of hemodynamics by participating in the formation of embryonic plasma. Nonetheless, according to the previous study of North et al. (2009), it is possible that knockdown of either *lrrc8aa* or *lrrc8ab* perturbed the extension of blood circulation to PBI and in turn affected definitive hematopoiesis (Fig. 3). Interestingly, LRRC8A was first found in a human patient with congenital agammaglobulinemia due to the lack of peripheral B cells (Sawada et al., 2003), whereas a later study in mice also indicates that the LRRC8A participates in the homeostasis of lymphocytes (Kumar et al., 2014). It is not understood whether or not these results are due to the early effect over the fate commitment of definitive hematopoiesis.

Initiation of brain ventricle inflation depends on Atp1a1a.1, a sodium-potassium pump that is thought to shape an osmotic gradient to drive fluid flux into the brain ventricle (Lowery and Sive, 2005). In this study, we showed that knockdown of either one of the *lrrc8a* paralogous genes also resulted in deflated brain ventricles (Figs 4 and 5), but most of the morphants were with initial formation of the brain ventricle. Therefore, it is likely that *lrrc8a* paralogs are required for continual expansion of the brain ventricle but not initial formation. It is proposed that the continual expansion of the brain ventricle might be dependent on blood circulation, as most of mutant zebrafish with brain ventricle phenotypes also have heart or circulation phenotypes (Schier et al., 1996). In line with this reasoning and as previously discussed, knockdown of either one of the *lrrc8a* paralogs resulted in the abnormality in cardiac and circulatory phenotypes. However, as both *lrrc8a* paralogs are expressed at ventricular walls and the cardiac output track, it is also possible that the expansion of both circulatory system and brain ventricle partly require common mechanisms such as VRAC and other ionic/osmotic regulatory mechanisms, and the sum of multiple mechanisms accounts for the complete morphogenesis. Hence the expansion of both spaces are affected when one or more of these mechanisms was defective.

Taurine efflux is considered one of the epitomic features of VRAC/VSOAC activities (Qiu et al., 2014; Voss et al., 2014). Although a volume-insensitive taurine efflux pathway via TauT is also proposed (Lambert et al., 2015), LRRC8A/LRRC8D constituted VRAC/VSOAC is the only channel that is proven to efflux intracellular taurine to the extracellular compartment (Planells-Cases et al., 2015). Our previous study showed that the homeostasis of taurine in zebrafish embryos predominantly depends on *de novo* synthesis via *csad* (Chang et al., 2013). Reduced *csad* level results in pericardial effusion, which is similar in *lrrc8a* morphants reported previously and in this study (Yamada et al., 2016) and can be rescued by taurine supplementation (Chang et al., 2013). Additionally, embryonic taurine deficiency also led to PBI accumulation (unpublished data) and a smaller brain ventricle (unpublished data). It is possible that both *lrrc8a* genes contribute to ventricular inflation via VRAC/VSOAC activity by modulating the distribution of organic osmolytes such as taurine. In line with this speculation, taurine supplementation ameliorated the smaller brain ventricle phenotype resulted from *lrrc8aa* and *lrrc8ab* knockdown (Fig. 6D,E).

Taken together, two zebrafish *lrrc8a* paralogous genes showed similar temporal and spatial expression patterns during early zebrafish embryogenesis, and contributed to the expansion of circulation and brain ventricle. It is likely that these two paralogs play redundant roles in brain ventricle expansion and organic osmolytes such as taurine contribute to this developmental procedure via VRAC/VSOAC.

## MATERIALS AND METHODS

### Zebrafish husbandry

The AB wild-type zebrafish were housed at a density of two to four fish per 3 l tank in the aquatic facility with an automatic recirculation system. The system was maintained at 28.5°C with a light/dark cycle of 14/10 h, and the fish were fed with live adult brine shrimp twice a day (Wei and Liu, 2014). Embryos were collected after spontaneous spawning, allowed to develop in E3 medium and staged by hpf at 28.5°C using morphological criteria (Kimmel et al., 1995). For the rescue experiment, embryos were cultured in E3 medium with or without the supplementation of taurine (Sigma Chemical Co.) as previously described (Chang et al., 2013). All experimental procedures in this study were reviewed and approved by the Institutional Animal Care and Use Committee of National Taiwan University (NTU104-EL-00085 and NTU105-EL-00147) and were performed in accordance with the approved guidelines.

### Molecular cloning

The total RNA of embryonic zebrafish was obtained as previously described (Chang et al., 2016). Briefly, 30 zebrafish embryos were homogenized in TRIzol Reagent (Life Technologies), mixed with 1-Bromo-3-chloropropane (Molecular Research Center) and then centrifuged at 12,000×***g*** for 15 min at 4°C. The aqueous phase was collected, mixed with 500 µl of isopropanol, briefly incubated and then centrifuged at 12,000×***g*** for 10 min at 4°C. The pellet was then washed with 75% ethanol, briefly air-dried and dissolved in DEPC-treated water. The single-stranded cDNA was synthesized from 2 µg of total RNA with random primers and High-Capacity cDNA Reverse Transcription Kit (Applied Biosystems, Foster City, CA, USA) or SuperScript III Reverse Transcriptase (Invitrogen). The resulting cDNAs were used both for cloning and RT-PCR.

To clone zebrafish *lrrc8aa* and *lrrc8ab*, primers were designed using the NEBuilder Assembly Tool (New England Biolabs), and the coding sequences of both genes were cloned with the Q5 Hot Start High-Fidelity 2X Master Mix (New England Biolabs). The sequences of primer pairs are listed in Table 1. The resulting DNA fragments were then cloned into pT7-IRES2-EGFP (Chang et al., 2013) with *Sal*I and *Bam*HI to give rise to pT7-*lrrc8aa*-IRES2-EGFP and pT7-*lrrc8ab*-IRES2-EGFP, respectively. After amplification (ECOS 101, Yeastern, Taipei, Taiwan) and purification (Presto Mini Plasmid Kit, Geneaid, Taipei, Taiwan), the sequences of cloned genes were confirmed by a sequencing service (Center for Biotechnology, National Taiwan University, Taipei, Taiwan).

**Table 1.**
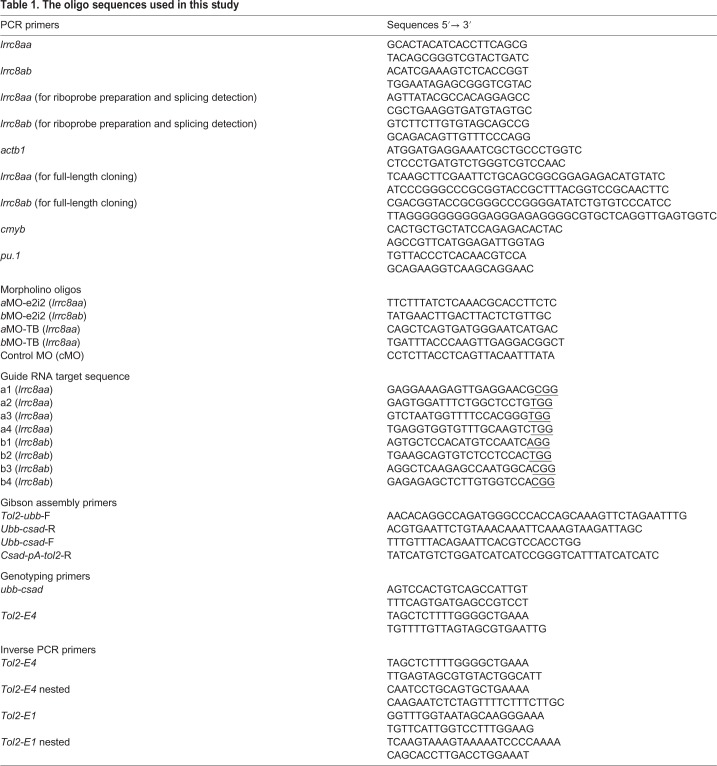
**The oligo sequences used in this study**

To construct a catalytically de-activated Cas9 (dCad9) that lacks endonucleolytic activity, pCS2-nzcas9n was used as template and two point mutations, D10A and H840A, were introduced into zebrafish optimized Cas9 to create the pCS2-nzdcas9n plasmid (Larson et al., 2013).

For expression, mRNA was synthesized using the mMESSAGE mMACHINE Kit (Ambion, Austin, TX, USA) after the pT7-IRES2-EGFP, pT7-*swell1a*-IRES2-EGFP, pT7-*swell1b*-IRES2-EGFP and pT7-*CSAD*2Δstop-FLAG-IRES2-EGFP (Chang et al., 2013) were linearized by *Afl*II, and pCS2-nzdcas9n was linearized by *Not*I. The synthesized mRNAs were aliquoted, stored at −80°C, and mixed with 0.05% phenol red immediately before use.

### qRT-PCR

To quantitatively analyze the expression levels of *lrrc8aa* and *lrrc8ab* and the hematopoietic differentiation markers, *cmyb* and *pu.1*, 4 µl of 10× diluted cDNA was mixed with 5 μl iQ SYBR Green Supermix (Bio-Rad) and 1 µl of primer set mix to amplify the fragments of target genes at 95°C for 3 min, and 39 cycles of 95°C for 3 s and 60°C for 30 s followed by 60°C for 1 min with a thermocycler (Thermo Fisher Scientific). The sequences of primer pairs are listed in Table 1.

### *In situ* hybridization

To demonstrate the spatial expression of zebrafish *lrrc8a* genes during embryonic development, whole-mount *in situ* hybridization was performed as described previously (Chang et al., 2013). Briefly, the DNA template for antisense digoxigenin-labeled riboprobes for *lrrc8aa* and *lrrc8ab* were generated by PCR and then synthesized by *in vitro* transcription using T7 polymerase. Zebrafish embryos were dechorionated, fixed with 4% paraformaldehyde in PBS and digested with proteinase K (10 μg/ml, Amresco, Solon, OH, USA) if older than 24 hpf. The embryos were then pre-hybridized for 3 h at 65°C without riboprobes and then hybridized with 50 ng RNA probe at 65°C overnight. After washing, hybridized embryos were blocked for 3 h at room temperature and incubated with Anti-Digoxigenin-AP Fab fragments (1:5000 in blocking solution; Roche Applied Science, Mannheim, Germany) with agitation at 4°C overnight. After washing, the hybridization signals were detected by NBT/BCIP solution (Roche Applied Science), observed and documented with a microscope (Leica Z16-APO).

### Gene knockdown by MO and CRISPR interference

To knockdown Lrrc8aa or Lrrc8ab, antisense MOs were designed against exon2-intron2 splicing sites or translation start site of respective genes (Fig. 1A) and a standard control morpholino was used as the control (Gene Tools LLC, Philomath, Oregon, USA). All MOs were dissolved in distilled water to make a 2 mM stock and diluted to desire concentration with 0.5% phenol red (Sigma Chemical Co.) before use.

The CRISPR interference (CRISPRi) was also used for gene-specific knockdown (Qi et al., 2013). A pool of guide-RNA (gRNA) targeting to non-template strands and near the transcriptional start sites of *lrrc8aa* and *lrrc8ab* was selected from CRISPRScan (Moreno-Mateos et al., 2015). A previously described gRNA-targeting eGFP was used as gRNA control (Shalem et al., 2014; Liao et al., 2015). Each gRNA was individually cloned into the *Bsm*BI site of pT7-sgRNA, sequenced and linearized by *Bam*HI for *in vitro* transcription using a MEGAshortscript T7 kit (Invitrogen). Microinjection was performed at the one-cell stage in embryos with 100 pg of *nzdcas9n* mRNA and 10 pg of gRNA mix per injection as previously described (Chang et al., 2013).

### Microangiography and heartbeat

For microangiography, TRITC-dextran (20 mg/ml) was injected into the sinus venosus of the anaesthetized [0.016% of ethyl 3-aminobenzoate methanesulfonate (MS-222, Sigma-Aldrich)] 28∼30 hpf zebrafish embryos, and the fluorescent images of the embryos were documented (Leica DM2500) at 32 hpf (Chen et al., 2006; Zhong et al., 2006). For the analysis of heart rate, 20 embryos of each treatment group were video-recorded under the microscope (Leica Z16-APO) and the heartbeat was manually counted in slow playback.

### Micrography and morphometrics for brain ventricle

To evaluate the inflation of the brain ventricle during embryogenesis, TRITC-dextran (20 mg/ml) was injected into the fourth ventricle of the anaesthetized 24 hpf zebrafish embryos, and the fluorescent images of the embryos were documented (Leica DM2500). For morphometric analysis, the area representing DMv (Fame et al., 2016) was manually depicted and calculated in ImageJ (Schneider et al., 2012).

### Generation of *csad* transgenic fishline

The zebrafish *ubiquitin B* (*ubb*) promoter (Mosimann et al., 2011) and *csad* coding sequence with FLAG tag (Chang et al., 2013) were cloned into *pDestTol2pA2* (Kwan et al., 2007) using Gibson Assembly Master Mix (New England Biolabs) resulting in *ubb:csadΔstop-FLAG* destination vector (Gibson et al., 2009). The primers used are listed in Table 1. One-cell-stage embryos were injected with 50 pg of DNA constructs and 50 pg of *transposase* mRNA. The injected embryos were raised and genotyped by fin-clipping upon adulthood. The founder fish carrying transgenes were out-crossed to wild-type fish to generate F1 fish. Adult F1 fish were genotyped and the integration sites were identified by inverse PCR (Kotani et al., 2006). In brief, the genomic DNA of F1 fish was digested with *Bgl*II/*BamH*1 or *Xba*I/*Nhe*I/*Avr*II/*Spe*I and ligated before undergoing nested PCR and sequencing. The *ubb:csadΔstop-FLAG* sequence was integrated in Chr24:28632092.28632099 (NC_007135.6) in the transgenic line used in this study. The F1 transgenic fish with the same integration site were inter-crossed to generate homozygous F2 transgenic fish Tg (*ubb:csadΔstop-FLAG*).

### Statistical analysis

The phenotypic penetrance, mortality, heart rate and gene expression level were subjected to Kruskal–Wallis test with Dunn's multiple comparisons. The area size of brain ventricle was analyzed by one-way ANOVA with Tukey's multiple comparisons test. All statistical analyses were performed using Prism 8 software (GraphPad), and *P*<0.05 was considered statistically significant. All data were presented as mean±s.e.m.
